# Genome sequence and description of *Aeromicrobium massiliense* sp. nov.

**DOI:** 10.4056/sigs.3306717

**Published:** 2012-11-15

**Authors:** Dhamodharan Ramasamy, Sahare Kokcha, Jean-Christophe Lagier, Thi-Thien Nguyen, Didier Raoult, Pierre-Edouard Fournier

**Affiliations:** Aix-Marseille Université, Faculté de médecine, Marseille, France

**Keywords:** *Aeromicrobium massiliense* genome

## Abstract

*Aeromicrobium massiliense* strain JC14^T^sp. nov. is the type strain of *Aeromicrobium massiliense* sp. nov., a new species within the genus *Aeromicrobium*. This strain, whose genome is described here, was isolated from the fecal microbiota of an asymptomatic patient. *Aeromicrobium massiliense* is an aerobic rod-shaped gram-positive bacterium. Here we describe the features of this organism, together with the complete genome sequence and annotation. The 3,322,119 bp long genome contains 3,296 protein-coding and 51 RNA genes.

## Introduction

*Aeromicrobium massiliense* strain JC14^T^ (= CSUR P158 = DSM 25782) is the type strain of *A. massiliense*. sp. nov. This bacterium is a motile, rod-shaped Gram-positive, aerobic catalase-positive bacterium that was isolated from the stool of a healthy Senegalese patient as part of a culturomics study aiming at cultivating all bacterial species within human feces [1].

Bacterial taxonomy, once reliant on “gold standards” such as DNA-DNA hybridization to delineate species and genera [2], has been significantly altered by the introduction of 16S rRNA amplification and sequencing [3]. The recent outcome of high throughput genome sequencing and proteomic analysis of bacteria adds a new and source of discriminative information, on which taxonomic proposals cn be based [4]. We proposed that this information be incorporated into a polyphasic approaches, as previously described [5], and used to describe new bacterial taxa [6,7].

The genus *Aeromicrobium* was created in 1991 [8] and consists of a group of related Gram-positive, aerobic, motile, rod-shaped bacteria. The genus *Aeromicrobium* belongs to the family *Nocardioidaceae* [9] within the order *Actinomycetales* [10]. *Aeromicrobium erythreum* was the first described species and is the type species of the genus *Aeromicrobium* [8]. In addition to this species, nine *Aeromicrobium* species have been validly published to date, including *A. alkaliterrae* [11], *A. fastidiosum* [12], *A. panaciterrae* [13], *and A. ginsengisoli* [14] that were *isolated* from soil; *A. marinum* [15], *A. tamlense* [16] *A. ponti* [17] and *A. halocynthiae* [18] that were recovered from marine environment; and *A. flavum* that was isolated from the air [19]. None of these species are reported to be human pathogens.

Here we present a summary classification and a set of features for *A. massiliense* sp. nov. strain JC14^T^ together with the description of the complete genomic sequencing and annotation. These characteristics support the circumscription of the species *A. massiliense*.

## Classification and features

A stool sample was collected from a healthy 16-year-old male Senegalese volunteer patient living in Dielmo (rural village in the Guinean-Sudanian zone in Senegal), who was included in a research protocol. Written assent was obtained from this individual. No written consent was needed from his guardians for this study because he was older than 15 years old (in accordance with the previous project approved by the Ministry of Health of Senegal and the assembled village population and as published elsewhere [20]). Both this study and the assent procedure were approved by the National Ethics Committee of Senegal (CNERS) and the Ethics Committee of the Institut Fédératif de Recherche IFR48, Faculty of Medicine, Marseille, France (agreement numbers 09-022 and 11-017). Several other new bacterial species were isolated from this specimen using various culture conditions, including the recently described *Anaerococcus senegalensis*, *Bacillus timonensis*, *Alistipes senegalensis*, *Alistipes timonensis*,*Clostridium senegalense*, *Peptoniphilus timonensis and Paenibacillus senegalensis* [6,7,21-25]. The fecal specimen was preserved at -80°C after collection and sent to Marseille. Strain JC14^T^ (Table 1) was isolated in December 2010 after inoculation on sheep blood-enriched Columbia agar (BioMérieux, Marcy l’Etoile, France), in 5% CO_2_ atmosphere at 37°C.

**Table 1:** Classification and general features of *Aeromicrobium massiliense* strain JC14^T^

**MIGS ID**	**Property**	**Term**	**Evidence code^a^**
	Current classification	Domain *Bacteria* Phylum *Actinobacteria* Class *Actinobacteria* Order *Actinomycetales* Family *Nocardioidaceae* Genus *Aeromicrobium* Species *Aeromicrobium massiliense* Type strain JC14^T^	TAS [26] TAS [27] TAS [28] TAS [10,28-30] TAS [9,28,30,31] TAS [8,11] IDA IDA
	Gram stain	positive	IDA
	Cell shape	rod-shaped	IDA
	Motility	motile	IDA
	Temperature range	mesophile	IDA
	Optimum temperature	25 - 37°C	IDA
MIGS-6.3	Salinity	growth in BHI medium + 5% NaCl	IDA
MIGS-22	Oxygen requirement	aerobic	IDA
	Carbon source	glucose, galactose, maltose, gluconate	NAS
	Energy source	chemoorganotrophic	NAS
MIGS-6	Habitat	human gut	IDA
MIGS-15	Biotic relationship	free living	IDA
MIGS-14	Pathogenicity Biosafety level Isolation	unknown 2 human feces	NAS
MIGS-4	Geographic location	Senegal	IDA
MIGS-5	Sample collection time	September 2010	IDA
MIGS-4.1	Latitude	13.7167	IDA
MIGS-4.2	Longitude	-16.4167	IDA
MIGS-4.3	Depth	surface	IDA
MIGS-4.4	Altitude	51 m above sea level	IDA

Evidence codes – IDA: Inferred from Direct Assay; TAS: Traceable Author Statement (i.e., a direct report exists in the literature); NAS: Non-traceable Author Statement (i.e., not directly observed for the living, isolated sample, but based on a generally accepted property for the species, or anecdotal evidence). These evidence codes are from the Gene Ontology project [32]. If the evidence is IDA, then the property was directly observed for a live isolate by one of the authors or an expert mentioned in the acknowledgements.

**Figure 1:**
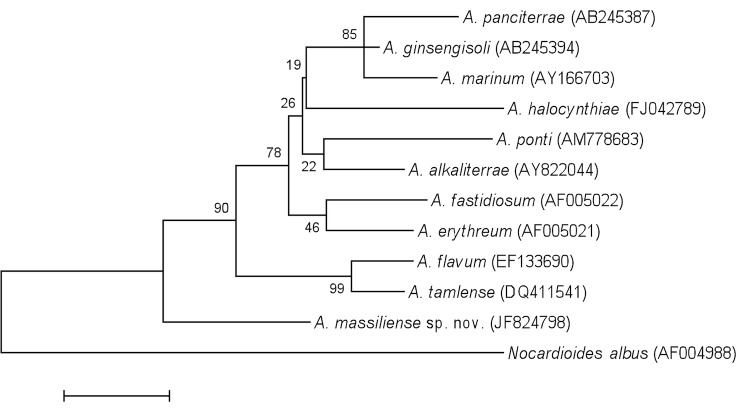
Phylogenetic tree highlighting the position of *Aeromicrobium massiliense* strain JC14^T^ relative to other type strains within the genus *Aeromicrobium*. GenBank accession numbers are indicated in parentheses. Sequences were aligned using CLUSTALW, and phylogenetic inferences obtained using the maximum-likelihood method within the MEGA software. Numbers at the nodes are bootstrap values obtained by repeating the analysis 500 times to generate a majority consensus tree. *Nocardioides albus* strain KCTC 9186 was used as outgroup. The scale bar represents a 1% nucleotide sequence divergence.

The strain exhibited nucleotide sequence similarities with validated *Aeromicrobium* species ranging from 95.37% with *A. marinum* (Bruns *et al*. 2003) [15] to 96.58% with *A. erythreum* (Miller *et al.* 1991) [8] (Figure 1), a value lower than the 98.7% 16S rRNA gene sequence threshold recommended by Stackebrandt and Ebers to delineate a new species without using DNA-DNA hybridization [3].

**Figure 2:**
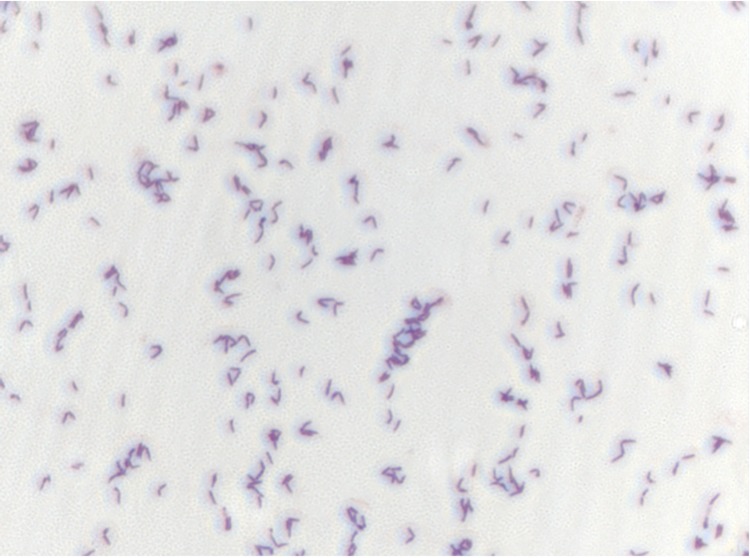
Gram staining of *A. massiliense* strain JC14^T^

**Figure 3:**
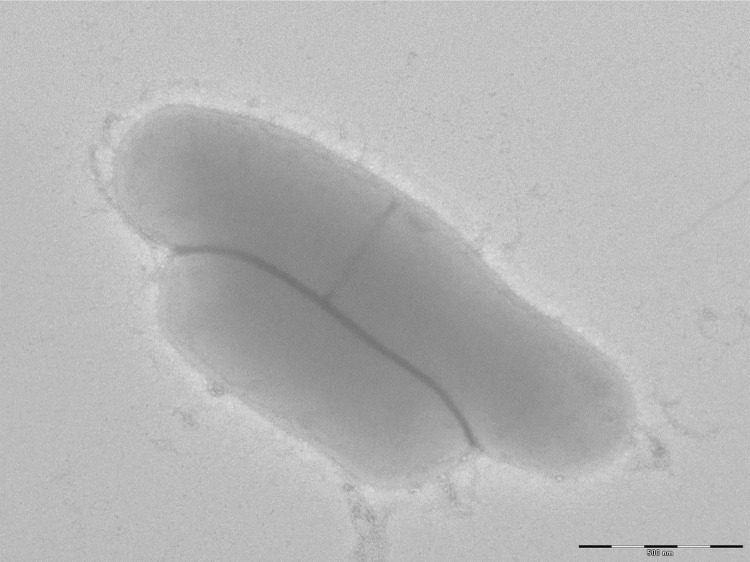
Transmission electron microscopy of *A. massiliense* strain JC14^T^ using a Morgani 268D (Philips) at an operating voltage of 60kV.The scale bar represents 500 nm.

Different growth temperatures (25, 30, 37, 45, 50°C) were tested; no growth occurred at 50°C, very weak growth occurred at 45°C, and optimal growth was observed between 25 to 37°C. Colonies were light yellow and opaque with a diameter of 1 mm on 5% blood-enriched Columbia agar (BioMérieux). Growth of the strain was tested under anaerobic and microaerophilic conditions using GENbag anaer and GENbag microaer systems, respectively (BioMérieux), and in the presence of air, with or without 5% CO_2_. Optimal growth was achieved under aerobic conditions, with or without CO_2_, and weak growth occurred in microaerophilic conditions. No growth was observed under anaerobic conditions. Gram staining showed rod-shaped Gram-positive bacteria. A motility test was positive. Cells grown on agar are Gram-positive (Figure 2) and have a mean diameter of 1.04 µm and a mean length of 1.67 µm (Figure 3).

Strain JC14^T^ exhibited catalase activity but not oxidase activity. Using API 20NE (Biomérieux), a positive reaction was obtained for nitrate reduction, aesculin and gelatin hydrolysis, glucose fermentation, β-galactosidase, maltose and gluconate assimilation. Other tested characteristics were negative. Using API ZYM (Biomérieux), a positive reaction was observed for α-glucosidase, β-glucosidase, trypsine, leucine arylaminidase, esterase lipase, and esterase. A weak reaction was observed for β- glucuronidase. Other tested characteristics were negative. *A. massiliense* is susceptible to penicillin G, amoxicillin, imipenem, and vancomycin but resistant to metronidazole. By comparison to *A. erythreum*, strain JC14^T^ differed in nitrate reduction, maltose assimilation, and susceptibility to penicillin G, amoxicillin and imipenem (Table 2) [8].

**Table 2:** Differential phenotypic characteristics of seven *Aeromicrobium* strains^†^.

	*A. massiliense*	*A. marinum*	*A. fastidiosum*	*A. alkaliterrae*	*A. flavum*	*A. erythreum*	*A. tamlense*
Cell morphology	Rods	Rods	Rods, cocci	Rods, cocci	Irregular rods	Irregular rods, cocci	Irregular rods
Motility	+	-	+	+	-	-	-
Catalase	+	+	+	+	+	+	+
Oxidase	-	+	+	-	-	-	-
Nitrate reduction	+	-	-	-	+	-	+
Indole production	-	-	-	-	-	-	-
Glucose fermentation	-	-	-	-	-	-	-
Urease	-	-	-	-	-	-	-
**Hydrolysis**							
Esculin	+	-	w	-	-	+	-
Gelatin	+	-	+	w	-	+	-
**Utilization of**							
Glucose	+	-	+	+	+	+	+
Arabinose	-	-	+	-	-	+	-
Mannose	-	-	-	-	-	-	-
Mannitol	-	-	-	-	-	-	-
N-actetyl-glucosamine	-	-	-	-	-	-	-
**API ZYM**							
Alkaline phosphatase	-	w	+	+	w	w	w
Esterase (C4)	+	w	+	na	w	w	w
Esterase lipase (C8)	+	+	+	+	w	+	+
Lipase (C14)	-	-	-	na	-	-	-
Leucine arylaminidase	+	+	+	+	+	+	+
Valine arylaminidase	w	-	w	-	-	-	+
Cystine arylaminidase	-	-	-	-	-	-	+
Acid phosphatase	-	-	+	+	-	+	+
Naphthol-AS-BI-phosphohydrolase	-	-	+	w	-	-	-
β-glucuronidase	w	-	-	-	-	-	-
α-glucosidase	+	-	+	+	+	+	+
β -glucosidase	+	-	-	-	w	-	w
DNA G+ C content	72.49	70.6	71-72	71.5	73.3	71-73	72.7
Isolated from	Human stools	German sea	Herbage	Alkaline soil	Air	Tropical soil	Dried seaweed

^†^*Aeromicrobium massiliense* strain JC14^T^, *Aeromicrobium marinum* strain DSM 15272^T^ *Aeromicrobium fastidiosum* strain DSM 10552^T^, *Aeromicrobium alkaliterrae* strain DSM 16824 ^T^, *Aeromicrobium flavum* strain DSM 19355 ^T^, *Aeromicrobium erythreum* strain DSM 8599^T^ and *Aeromicrobium tamlense* strain DSM 19087^T^.

**Figure 4:**
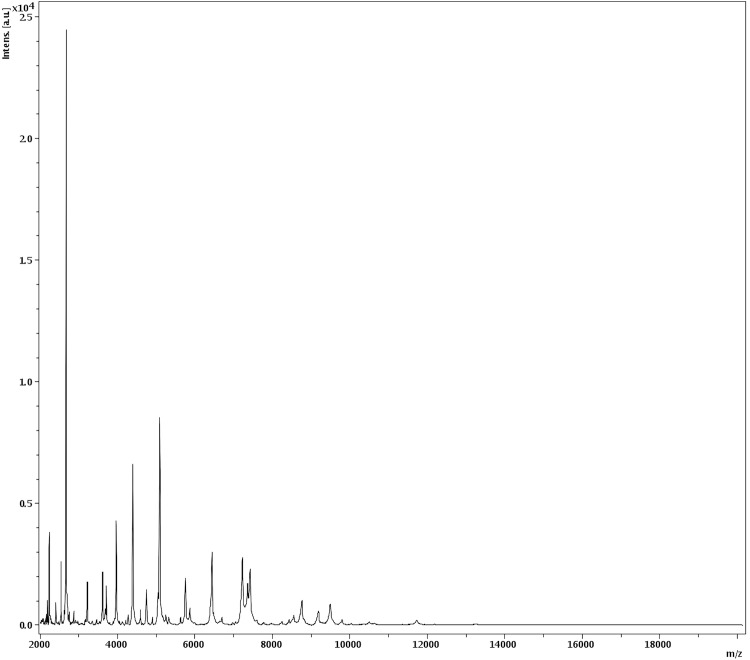
Reference mass spectrum from *A. massiliense* strain JC14^T^. Spectra from 12 individual colonies were compared and a reference spectrum was generated.

Matrix-assisted laser-desorption/ionization time-of-flight (MALDI-TOF) MS protein analysis was carried out as previously described [7,33] using a Microflex spectrometer (Bruker Daltonics, Germany). Twelve distinct deposits were done for strain JC14^T^ from 12 isolated colonies. The twelve JC14^T^ spectra were imported into the MALDI BioTyper software (version 2.0, Bruker) and analyzed by standard pattern matching (with default parameter settings) against the main spectra of 3,769 bacteria, which were used as reference data, in the BioTyper database. The database contained no spectra from validly published *Aeromicrobium* species. No significant score was obtained for strain JC14^T^, thus suggesting that our isolate was not a member of a known species within the Bruker database. However, we acknowledge that the absence of other *Aeromicrobium* spectra does not as yet make using MALDI TOF MS a discriminatory identification criterion for *A. massiliense*. We incremented our database with the spectrum from strain JC14^T^ (Figure 4).

## Genome sequencing and annotation

### Genome project history

The organism was selected for sequencing on the basis of its phylogenetic position and 16S rRNA similarity to other members of the genus *Aeromicrobium*.It is part of a study of the human digestive microbiota aimed at isolating all bacterial species found in human feces. It was the second genome of an *Aeromicrobium* species and the first genome of *Aeromicrobium massiliense* sp. nov. The genome EMBL accession number is CAHG00000000 and consists of 18 contigs. Table 3 shows the project information and its association with MIGS version 2.0 compliance [34].

**Table 3:** Project information

**MIGS ID**	**Property**	**Term**
MIGS-31	Finishing quality	High quality draft
MIGS-28	Libraries used	Shot Gun, Paired-end 3 Kb
MIGS-29	Sequencing platforms	454
MIGS-31.2	Fold coverage	77 ×
MIGS-30	Assemblers	Newbler version 2.5.3
MIGS-32	Gene calling method	Prodigal
	EMBL ID	CAHG00000000
	Genbank Date of Release	June 1, 2012
	Project relevance	Study of the human gut microbiot

### Growth conditions and DNA isolation

*A. massiliense* sp. nov. strain JC14^T^ (CSUR P158, DSM 25782) was grown aerobically on 5% sheep blood-enriched blood agar at 37°C. Four petri dishes were spread and resuspended in 3x100µl of G2 buffer (EZ1 DNA Tisue kit, Qiagen). A first mechanical lysis was performed using glass powder on a Fastprep-24 device (Sample Preparation system; MP Biomedicals, USA) using 2×20 seconds. DNA was then treated with 2.5µg/µL lysozyme (30 minutes at 37°C) and extracted through a BioRobot EZ 1 Advanced XL (Qiagen).The DNA was then concentrated and purified on a Qiamp kit (Qiagen). The yield and the concentration was measured by the Quant-it Picogreen kit (Invitrogen) on a Genios Tecan fluorometer at 90 ng/µl.

### Genome sequencing and assembly

A shotgun and a 3 kb paired-end sequencing strategies were used (Roche). Both libraries were pyrosequenced on the GS FLX Titanium sequencer (Roche). This project was loaded on a 1/4 region of PTP Picotiterplate (Roche, Meylan, France) for the shotgun library and 2 ×1/8 region for the 3-kb paired-end library. The shotgun library was constructed with 500ng of DNA with the GS Rapid library Prep kit as described by the manufacturer (Roche). For the paired-end library, 5µg of DNA was mechanically fragmented on a Hydroshear device (Digilab, Holliston, MA, USA) with an enrichment size at 3-4kb. DNA fragmentation was visualized through an Agilent 2100 BioAnalyzer on a DNA labchip 7500 with an optimal size of 3.428kb. The library was constructed according to the 454 Titanium paired-end protocol (Roche). Circularization and nebulization were performed and generated a pattern with an optimal at 367bp. After PCR amplification through 15 cycles followed by double size selection, the single stranded paired-end library was then quantified on usingQuant-it Ribogreen (Invitrogen) on a Genios Tecan fluorometer at 175pg/µL. The library concentration equivalence was calculated at 8.75E+08 molecules/µL. The libraries were stored at -20°C until further use.

The shotgun library was clonally amplified with 3 cpb in 4 emPCR reactions and the 3-kb paired-end library was amplified with 0.5, 0.75 and 1 cpb in 2 emPCR reactions with the GS Titanium SV emPCR Kit (Lib-L) v2 (Roche). The yield of the shotgun emPCR reactions was 13%, and the yields of the paired-end emPCRs were 9.1, 10.8, and 9.5% for the 0.5, 0.75 and 1 cpb conditions, respectively, in the range of 5 to 20% from the Roche procedure.

Approximately 790,000 and 340,000 beads for the shotgun and paired-end libraries, respectively, were loaded on the GS Titanium PicoTiterPlate PTP Kit 70×75 and sequenced with the GS FLX Titanium Sequencing Kit XLR70 (Roche). The runs were performed overnight and then analyzed on the cluster through the gsRunBrowser and Newbler Assembler (Roche). A total of 322,810 and 108,529 passed filter wells were obtained for the shotgun and paired-end strategies, respectively, and generated 122.9 and 33.2 Mb with length averages of 381 and 306 bp, respectively. The passed filter sequences were assembled using Newbler with 90% identity and 40 bp as overlap. The final assembly identified 18 contigs (>1,500bp) arranged into 5 scaffolds and generated a genome size of 3.32 Mb.

### Genome annotation

Open Reading Frames (ORFs) were predicted using Prodigal [35] with default parameters but the predicted ORFs were excluded if they were spanning a sequencing GAP region. The predicted bacterial protein sequences were searched against the GenBank database [36] and the Clusters of Orthologous Groups (COG) databases using BLASTP. The tRNAscan-SE tool [37] was used to find tRNA genes, whereas ribosomal RNAs were found by using RNAmmer [38]. Transmembrane domains and signal peptides were predicted using TMHMM [39] and SignalP [40], respectively. ORFans were identified if their BLASTp *E-*value was lower than 1e-03 for alignment length greater than 80 amino acids. If alignment lengths were smaller than 80 amino acids, we used an *E*-value of 1e-05. Such parameter thresholds have been used in previous works to define ORFans. To estimate the mean level of nucleotide sequence similarity at the genome level between *A. massiliense* and *A. marinum* (GenBank accession number ACLF00000000), the only available *Aeromicrobium* genome to date, we compared the ORFs only using BLASTN at a query coverage of ≥ 70% and a minimum nucleotide length of 100 bp.

## Genome properties

**Figure 5:**
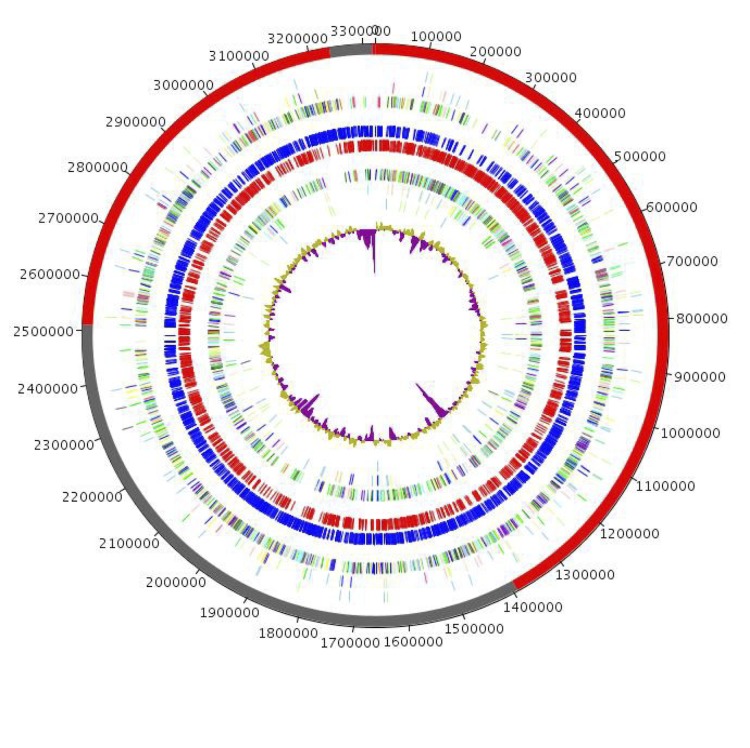
Graphical circular map of the *A. massiliense* strain JC14^T^ genome. From outside to the center: scaffolds (red / grey), COG category of genes on the forward strand (three circles), genes on forward strand (blue circle), genes on the reverse strand (red circle), COG category on the reverse strand (three circles), G+C content.

The genome is 3,322,119 bp long (1 chromosome, but no plasmid) with a 72.49% G+C content (Table 4 and Figure 5). Of the 3,347 predicted genes, 3,296 were protein-coding genes, and 51 were RNAs (1 rRNA operon, 2 addition 5S rRNAs, and 46 tRNAs).

**Table 4:** Nucleotide content and gene count levels of the genome

**Attribute**	**Value**	**% of total^a^**
Genome size (bp)	3,322,119	100
DNA coding region (bp)	3,109,049	93.59
DNA G+C content (bp)	2,408,297	72.49
Total genes	3,347	100
RNA genes	51	1.52
Protein-coding genes	3,296	98.48
Genes with function prediction	2,358	71.54
Genes assigned to COGs	2,334	70.81
Genes with signal peptides	501	15.2
Genes with transmembrane helices	778	23.6

^a^ The total is based on either the size of the genome in base pairs or the total number of protein coding genes in the annotated genome

A total of 2,358 genes (71.54%) were assigned a putative function. In addition, 292 genes were identified as ORFans (8.86%). The remaining genes were annotated as hypothetical proteins. The distribution of genes into COGs functional categories is presented in Table 5. The properties and the statistics of the genome are summarized in Tables 4 and 5.

**Table 5:** Number of genes associated with the 25 general COG functional categories

**Code**	**Value**	**%age**^a^	**Description**
J	159	4.82	Translation
A	0	0	RNA processing and modification
K	223	6.77	Transcription
L	112	3.4	Replication, recombination and repair
B	2	0.06	Chromatin structure and dynamics
D	21	0.64	Cell cycle control, mitosis and meiosis
Y	0	0	Nuclear structure
V	81	2.45	Defense mechanisms
T	155	4.7	Signal transduction mechanisms
M	93	2.82	Cell wall/membrane biogenesis
N	37	1.12	Cell motility
Z	1	0.03	Cytoskeleton
W	0	0	Extracellular structures
U	38	1.15	Intracellular trafficking and secretion
O	84	2.55	Posttranslational modification, protein turnover, chaperones
C	149	4.52	Energy production and conversion
G	175	5.3	Carbohydrate transport and metabolism
E	267	8.1	Amino acid transport and metabolism
F	56	1.7	Nucleotide transport and metabolism
H	119	3.61	Coenzyme transport and metabolism
I	131	3.7	Lipid transport and metabolism
P	164	4.98	Inorganic ion transport and metabolism
Q	97	2.94	Secondary metabolites biosynthesis, transport and catabolism
R	376	11.41	General function prediction only
S	183	5,55	Function unknown
-	962	29.19	Not in COGs

^a^ The total is based on the total number of protein coding genes in the annotated genome

### Genome comparison

To date, the genome from *A. marinum* strain DSM15272 is the only genome from *Aeromicrobium* species that has been sequenced. By comparison with the draft genome of *A. marinum*, *A. massiliense* had a larger genome (3.32 *vs* 2.58Mb, respectively), a higher G+C content (72.5 *vs* 70.6%) but a smaller number of predicted genes (3,347 *vs* 3,625 genes). In addition, *A. massiliense* and *A. marinum* shared a mean nucleotide sequence similarity of 77.32% at the genome level (range 70.42 – 100%).

## Conclusion

On the basis of phenotypic, phylogenetic and genomic analyses, we formally propose the creation of *Aeromicrobium massiliense* sp. nov. that contains the strain JC14^T^. This bacterium has been detected in Senegal.

### Description of *Aeromicrobium massiliense* sp. nov.

*Aeromicrobium massiliense* (ma.si.li.e’n.se L.gen. neutr. adj. massiliense, of Massilia, the latin name of Marseille, where was isolated *A. massiliense*).

Colonies are light yellow and opaque with a diameter of 1 mm on blood-enriched Columbia agar. Cells are rod-shaped with a mean diameter of 1.04 µm. Optimal growth is achieved obtained aerobically with or without CO_2_ or under microaerophilic conditions. No growth is observed under anaerobic conditions. Growth occurs between 25 - 37°C. Cells stain Gram-positive, are non-endospore forming, and motile. Catalase, nitrate reduction, aesculin and gelatin hydrolysis, glucose fermentation, β-galactosidase, maltose and gluconate assimilation, α-glucosidase, β-glucosidase, β- glucuronidase, trypsine, leucine arylaminidase, esterase lipase, and esterase activities are present. Oxidase activity is absent. Cells are susceptible to penicillin G, amoxicillin, imipenem, and vancomycin but resistant to metronidazole. The G+C content of the genome is 72.49%. The 16S rRNA and genome sequence are deposited in EMBL under accession numbers JF824798 and CAHG00000000, respectively. The type strain JC14^T^ (= CSUR P158 = DSM 25782) was isolated from the fecal microbiota of a healthy patient in Senegal.
